# A Monte Carlo study of high-energy photon transport in matter: application for multiple scattering investigation in Compton spectroscopy

**DOI:** 10.1107/S1600577515018603

**Published:** 2016-01-01

**Authors:** Marek Brancewicz, Masayoshi Itou, Yoshiharu Sakurai

**Affiliations:** a Japan Synchrotron Radiation Research Institute (JASRI), 1-1-1 Kouto, Sayo-cho, Sayo-gun, Hyogo 679-5198, Japan

**Keywords:** photon transport, multiple scattering, Monte Carlo, Compton spectroscopy, simulation algorithm

## Abstract

The first results of multiple scattering simulations of polarized high-energy X-rays for Compton experiments using a new Monte Carlo program, *MUSCAT*, are presented. The program is developed to follow the restrictions of real experimental geometries. The new simulation algorithm uses not only well known photon splitting and interaction forcing methods but it is also upgraded with the new propagation separation method and highly vectorized. In this paper, a detailed description of the new simulation algorithm is given. The code is verified by comparison with the previous experimental and simulation results by the ESRF group and new restricted geometry experiments carried out at SPring-8.

## Abstract 2

The new efficient Monte Carlo procedure for multiple scattering simulation in Compton experiments is presented. The simulation algorithm has proved its advantage in restricted geometry experiments.

## 1. Introduction   

Compton scattering is a well established experimental method for investigating the electronic properties of matter (Williams, 1977 ▸; Cooper *et al.*, 2004 ▸). It measures the intensity of radiation scattered on a sample in the solid angle dΩ in energy intervals d
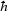
ω (double differential cross section):
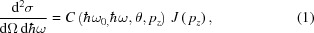
where *C* is a certain function depending on the following scattering parameters: incident photon energy (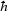
ω_0_), scattered photon energy (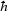
ω), scattering angle (θ), electron momentum along the scattering vector (*p_z_*). *J*(*p*_*z*_) is the one-dimensional projection of the three-dimensional electron momentum density [ρ(**p**)] onto the scattering vector and is called the Compton profile:
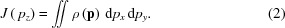
Extraction of the Compton profile from experimental spectra is a standard procedure including a series of energy- and geometry-dependent corrections. However, there are still small but significant discrepancies observed between theoretical and experimental Compton profiles even in high-momentum regions, where theoretical description of core electrons should work well (Brancewicz *et al.*, 2013 ▸). There are two possible sources of this discrepancy: one is the uncertainty associated with the experimental background measurements and the other is the influence of multiple-scattered photons (MSC) in experimental spectra. Experimental backgrounds can be reduced, for example, by using a special sample holder, vacuum chamber, transmitted beam absorbers, collimators and additional absorbing shields. The effect of MSC can be reduced only by using thinner samples, but this leads to a significant reduction of single Compton scattering intensities.

The spectral shape of MSC distorts the obtained electron momentum density distribution. The influence of multiple scattering can be cancelled if the difference in Compton profiles are considered, for example the difference between directional profiles measured on two samples of the same size. Apart from the case where the difference profiles are measured on one sample but under different conditions (temperature, pressure), the effect of MSC does not completely vanish, and the accurate simulation of the MSC spectrum can be crucial to obtain the high-quality Compton profiles as well as the difference profiles. Therefore, the simulation of MSC spectra is crucial in Compton scattering experiments.

## 2. Monte Carlo procedures   

The problem of finding the spectral distribution of multiple-scattered X-rays by analytical calculations has been considered many times (Dumond, 1930 ▸; Williams *et al.*, 1974 ▸; Tanner & Epstein, 1976*a* ▸,*b* ▸,*c* ▸; Braun-Keller & Epstein, 1977*a* ▸,*b* ▸; Das *et al.*, 1988 ▸). However, analytical approaches are very difficult to adopt for the real experimental geometry (beam size, sample shape, collimators) and are limited only to double scattering. Therefore, the Monte Carlo (MC) simulation is the most appropriate method of finding the MSC contribution to the measured Compton profile and has been used in Compton spectroscopy.

**Figure 1:**
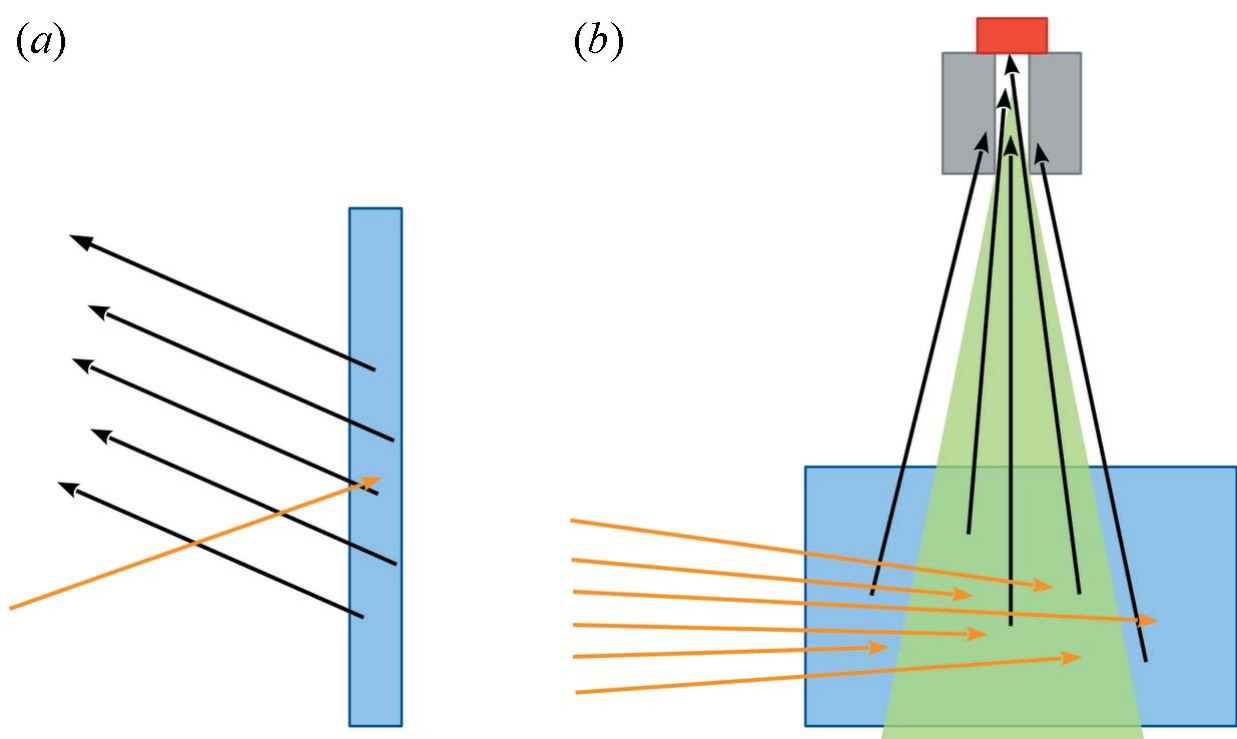
Difference between relaxed (*a*) and restricted (*b*) geometries used in Monte Carlo simulations for multiple X-ray scattering. Orange arrows are the incident beam, black arrows show the directions of the scattered beam. The detection area (seen by detector) is marked by the transparent green colour. The detector (red) is placed after the collimator (grey).

The first Monte Carlo procedure for multiple scattering simulations of unpolarized γ-rays in Compton scattering experiments was described (Felsteiner *et al.*, 1974 ▸) and experimentally tested (Felsteiner & Pattison, 1975 ▸) 40 years ago. It uses solutions proposed by Cashwell & Everett (1959 ▸) in order to improve the simulation efficiency by applying the idea of forcing the respective processes and the corresponding photon weight reduction (photon splitting). This procedure does not take into account the incident beam size nor the collimation of scattered beam; all photons scattered into a specific direction are considered as registered. This approximation is called a relaxed geometry (see Fig. 1*a* ▸). There are also attempts to adapt this code to the individual experimental setup, taking into account the size and divergence of the incident beam as well as collimation of scattered beam (restricted geometry, Fig. 1*b* ▸), and the simulation results are significantly different from the relaxed geometry (Itoh *et al.*, 1979 ▸).

The Monte Carlo procedure mentioned above has not been tested experimentally in a direct way. The only criterion for its accuracy used so far is the degree of agreement between obtained Compton profiles and theoretical predictions (Felsteiner & Pattison, 1975 ▸). Despite the noticeable improvement of final experimental Compton profiles after the MSC correction, the authors have suggested that the results are still insufficient and one should always strive to minimize multiple scattering by performing measurements on samples as thin as possible (Felsteiner *et al.*, 1974 ▸; Felsteiner & Pattison, 1975 ▸). The first attempt for the direct experimental verification of Felsteiner’s procedure was made by Pitkanen *et al.* (1986 ▸). The experimental results are consistent with the simulation results, although the simulation statistics are poor.

The first improvement of the Monte Carlo procedure (scattering forcing and photon splitting) for linearly polarized photons of a synchrotron beam was made by Chomilier *et al.* (1985 ▸). Implementation of circular beam polarization and magnetic inelastic scattering have been carried out by Sakai (1987 ▸) using the formulas derived by Lipps & Tolhoek (1954 ▸). Despite the good reproduction of magnetic Compton profiles (Kakutani & Sakai, 2004 ▸), this program has not been tested in a direct way for the reproduction of the multiple scattering part of the simulated spectrum.

The same method (scattering forcing and photon splitting) has been used in the Monte Carlo procedure developed at ESRF (Fajardo *et al.*, 1998 ▸). The procedure was experimentally tested for near relaxed geometry. The comprehensive and successful verification by the authors makes this procedure the most accurate for multiple scattering simulations. We will use their results to verify our new program *MUSCAT* in the relaxed geometry approximation.

## 3. *MUSCAT* – the new Monte Carlo algorithm   

**Figure 2:**
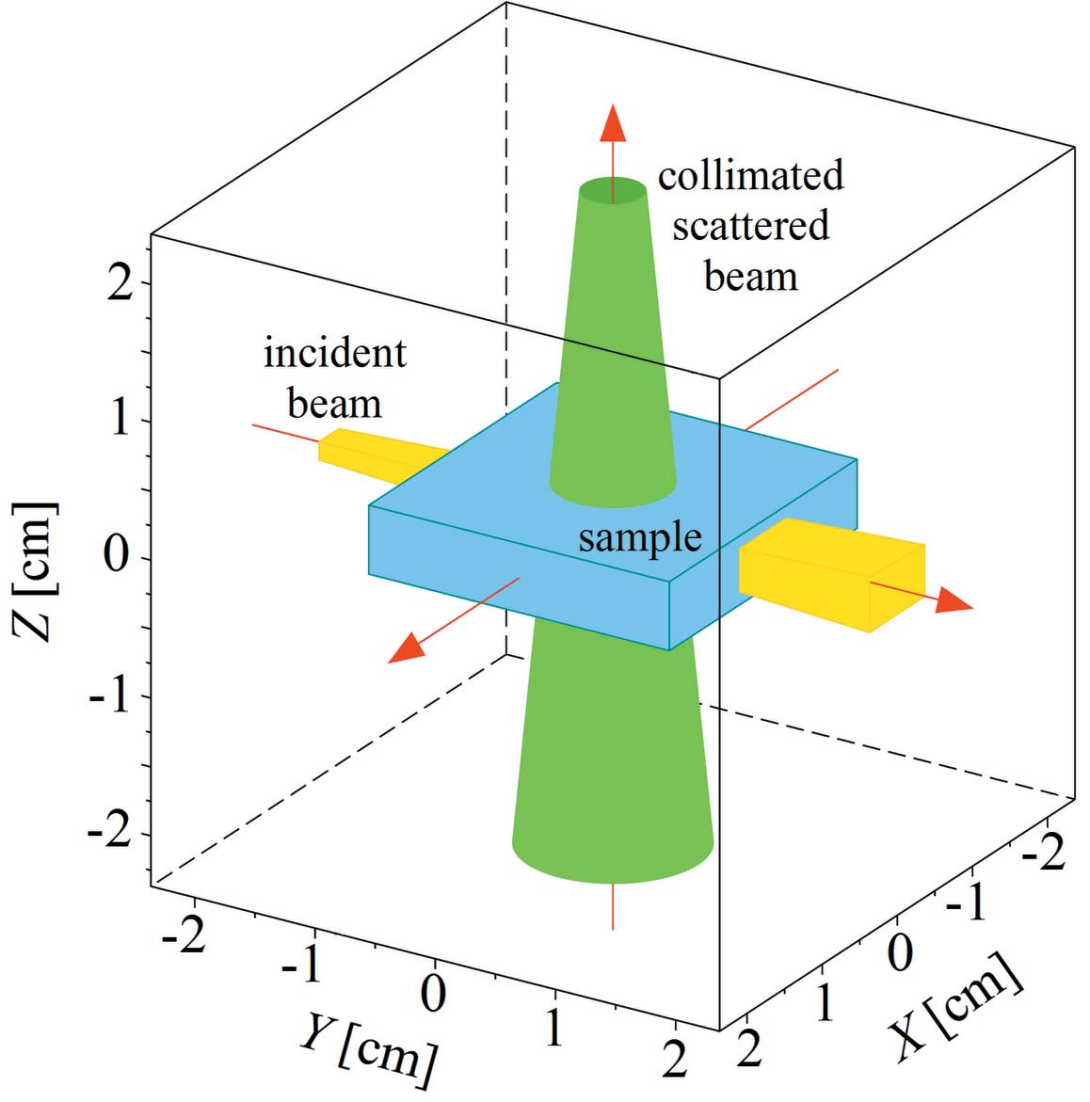
Sample geometry realised in the *MUSCAT* program. The incident beam (yellow) propagates along the *Y*-axis. The sample is drawn in blue. The green colour shows the detection area formed by the collimator shape. Each photon that passes through the collimator is treated as detected.

The main task of the newly developed procedure is to deal with the highly restrictive geometry and large samples like Li-ion batteries in Compton scattering imaging experiments (Itou *et al.*, 2015 ▸), where the relaxed geometry of MSC simulation does not work. The layered structure of such samples should be implemented after the successful verification of the code for a homogeneous material sample, which is presented in this paper. One of the main program objectives is to deal with variations of experimental geometries for future flexible modifications. An example of highly restricted geometry presenting the program possibilities is shown in Fig. 2 ▸. Other experimental geometries can be realised through the series of transformations: translations, rotations, resizing and positioning of every element (beam, sample, collimator). In its present state, the program is only limited to cuboid sample shapes, but other shapes (*e.g.* cylindrical, spherical) could also be introduced.

**Figure 3:**
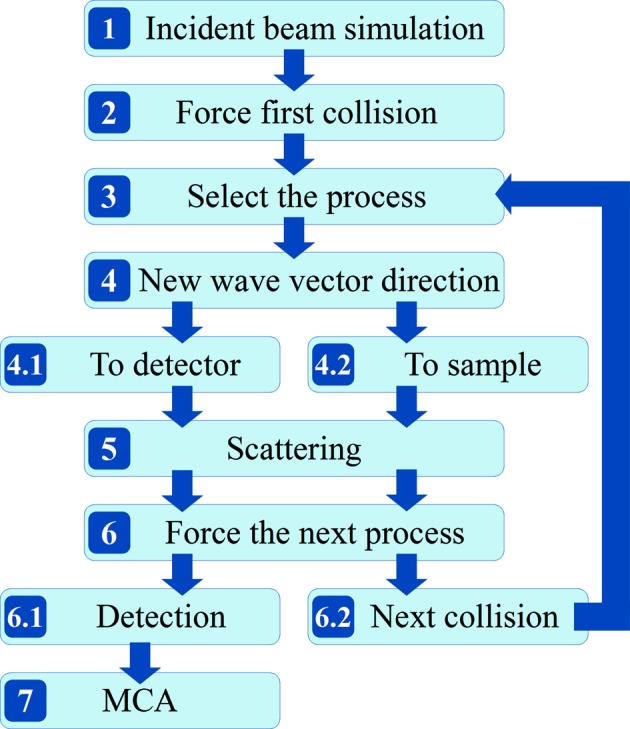
Block algorithm for multiple scattering simulations used by the *MUSCAT* program. Loop 4 → 4.2 → 5 → 6 → 6.2 → 3 can be repeated for the second, third and more orders of scatterings.

In the *MUSCAT* code the method of scattering forcing and photon splitting has been highly extended. Together with a new way of treating scattered photons separately for detection and next scattering we have improved the simulation efficiency. This means the number of registered events (weighted parts of photons) is the same as the number of incident photons at the sample (if the detector is not collimated) for every order of scattering considered. The new improved Monte Carlo algorithm is shown in Fig. 3 ▸ and described in detail below.

### 3.1. Incident beam simulation   

Information about the incident beam is stored in a table of photon data (beam matrix), with initial weights *W*_0_ = 1. The shape of the initial beam is defined by the given size of the source and the slit between the source and sample. The direction of photon propagation (wavevector) is defined by two randomly chosen points, one in the source and one in the slit. The components of the initial electric vectors in a plane perpendicular to the wavevectors are calculated from:
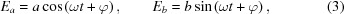
at the time *t* = 0, and phase φ chosen by uniform random sampling (incoherent beam). The semi-major (*a*) and semi-minor (*b*) axes lengths of the polarization ellipse are calculated from the linear polarization Stokes parameter *P*_1_ as an input:
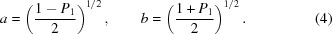
The simulated beam propagates from the source to the sample surface along the wavevectors.

### 3.2. Force first collision   

A photon that hits the sample surface is forced to interact with matter in the sample volume by avoiding the transmission process. In this case the photon is propagated by distance *l* (penetration depth) given by:
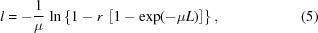
where μ is the total attenuation coefficient, *L* is the distance from the current photon position on the sample surface to the exit point from the sample (optical thickness), and *r* is a random number between 0 and 1. Due to the probability *P*_t_ of the transmission process, the photon weight is reduced from *W*_0_ = 1 to *W*:
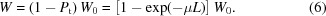
After the weight reduction from *W*_0_ to *W*, to avoid multiplication of variables, weight *W* become *W*_0_ again before the next considered process that also involves the weight reduction.

### 3.3. Select the process   

In the case of Compton scattering experiments, the typical incident photon energy is from 59.54 keV (^214^Am isotope source) to 662 keV (^137^Cs isotope source). Three types of interaction with matter dominate over the energy range: photoelectric absorption, elastic scattering and inelastic scattering. As long as we are dealing with light elements, whose emission lines are below the energy scale of the considered spectra, we can force only elastic and inelastic scattering by a corresponding weight reduction due to the probability *P*_p_ of the photoelectric absorption process:
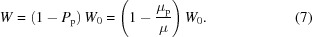
The probability of photoelectric absorption is defined as the ratio between the photoelectric (μ_p_) and total (μ) attenuation coefficients. Elastic or inelastic process selection is then carried out through the random sampling with probability proportional to the appropriate attenuation coefficients; μ_e_ for elastic and μ_i_ for inelastic scattering.

### 3.4. New wavevector direction   

From this point the simulation process differs from the old procedures. The beam matrix is dealt with in two independent ways. One is the scattering direction towards the detector (algorithm step 4.1 in Fig. 3 ▸) where the new wavevectors are selected by pointing the detector direction (collimator exit) randomly into the solid angle of the detector Ω_d_. The other is random scattering into the sample volume (algorithm step 4.2 in Fig. 3 ▸) where the new wavevectors directions are selected by random sampling into the full solid angle 4π. We called this method propagation separation and it is crucial for improving the simulation efficiency, because it allows the preservation of the total number of photons (their weighted parts) regardless of the considered propagation direction (to the detector or random) and scattering order. New electric vectors are obtained by rotation of initial electric vectors around the normal to the scattering plane by an angle α equal to the angle between the normals to the incident and scattering planes. Angle Θ between the incident and scattered electric vectors is calculated.

### 3.5. Scattering   

The energy of elastically scattered photons does not change. In the case of inelastic scattering, the scattered photon energy 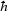
ω is calculated based on the scattering angle θ and incident energy 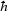
ω_0_:
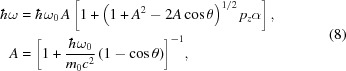
where α is the fine structure constant and *m*_0_ *c* ^2^ is the electron rest mass (in keV). The *z*-component of electron momentum *p*_*z*_ [in atomic units (a.u.)] is selected by random sampling with frequency proportional to the given Compton profile distribution *J*(*p*_*z*_).

Weight reduction resulting from the differential scattering cross section is then made:
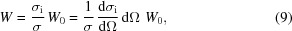
where σ_i_ is the individual photon scattering cross section, σ is the total cross section, dσ_i_/dΩ is the individual differential cross section for inelastic (Compton) scattering on an atom [dσ_C_/dΩ, equation (10)] or elastic (Thomson) scattering [dσ_R_/dΩ, equation (12)]. The solid angle dΩ depends on the considered type of propagation and equals Ω_d_ (detector solid angle) in the case of scattering towards the detector or 4π if random scattering into the whole solid angle is considered.

The differential cross section for inelastic scattering on an atom is calculated using a combination of the Klein–Nishina formula for a single electron (Tanner & Epstein, 1976*a* ▸) and non-relativistic Hartree–Fock atomic incoherent scattering functions *S*(*x*′, *Z*) (Hubbell *et al.*, 1975 ▸):
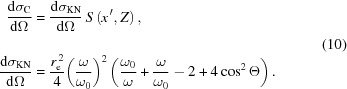
In the case of energy transfer lower than the electron’s binding energy, inelastic scattering does not occur. Additional correction for this effect can be made by a weight reduction resulting from the probability of inelastic scattering on electrons from a corresponding shell (*X*):
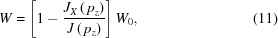
where *J*_*X*_(*p*_*z*_) is a value of the *X* shell Compton profile and *J*(*p*_*z*_) is a value of the total Compton profile for selected momentum component *p*_*z*_.

The differential cross section for elastic scattering on an atom (Rayleigh scattering), dσ_R_/dΩ, is calculated using a combination of the Thomson formula dσ_T_/dΩ (Tanner & Epstein, 1976*c* ▸) and relativistic Hartree–Fock atomic form factors *F*(*x*, *Z*) (Hubbell & Overbo, 1979 ▸):
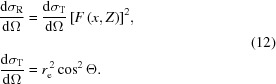
In equations (10) and (12), Θ is the angle between the incident and final (after scattering) polarization states, *r*_e_ is the classical electron radius, *x* and 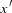 are the momentum transfer parameters for coherent and incoherent scattering, respectively (Hubbell *et al.*, 1975 ▸).

### 3.6. Force the next process   

After the scattering, the next process is forced depending on the previous history of the photons. Photons scattered into the detector direction (algorithm step 4.1) are forced to be detected (algorithm step 6.1). Photons scattered into the sample volume (algorithm step 4.2) are forced to the next collision within the sample volume (algorithm step 6.2).

#### 3.6.1. Detection   

Photons scattered towards the detector are forced to leave the sample (transmission process). Appropriate weight reduction due to the rejection of any interaction processes inside the sample is applied:
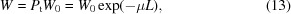
where *P*_t_ is the probability of the transmission process and *L* is the photon distance to the exit point from the sample. A photon propagating to the detector and passing through the collimator is considered as a detected event.

#### 3.6.2. Next collision   

After the scattering all photons are forced into a second interaction within the sample volume exactly like in algorithm step 2, with appropriate weight reduction due to the transmission process rejection [equation (6)]. In order to simulate the next scattering process one should go to algorithm step 3.

### 3.7. Multichannel analyser   

Multichannel analyser (MCA) is a group of procedures that are designed to calculate the final partial spectra of single, double, triple and more scattering orders from the matrix of detected photons. It also contains the procedure for final spectra convolution to the desired experimental resolution and calculation of some final simulation parameters.

All of the total cross sections (σ) and mass attenuation coefficients (μ) for inelastic scattering, elastic scattering and photoelectric absorption used in the *MUSCAT* simulation program were calculated using the *XCOM* program (version 3.1; 23 June 1999) from NIST (Berger & Hubbel, 1987 ▸), also available as an on-line interactive database (Berger *et al.*, 2010 ▸).

## 4. Experimental verification   

First simulation results using *MUSCAT* have been performed for the Compton experiment geometry widely used at SPring-8 (beamline BL08W) with an incident energy of 115.6 keV, a scattering angle of 165° and a sample of 2 mm-thick Al. The double to single scattering spectra intensity ratio within the range of a Compton profile from −10 to 10 a.u. (double scattering level) is estimated, based on the simulated spectra, to be around 10%. The value obtained completely disagrees with the simulation results by the codes of Sakai or Felsteiner (about 3%). Therefore a robust experimental verification of the simulation results is needed.

We think that the best and most comprehensive method for experimental verification of the MSC simulation procedure is the one presented by the group from ESRF (Fajardo *et al.*, 1998 ▸). It consists of three different experiments with the use of highly linearly polarized (in the scattering plane) 57.8 keV synchrotron X-rays and a set of different Al samples. All experiments are described in detail in the cited work. Together with the experimental data they show the corresponding Monte Carlo simulation results made by their own procedure. An excellent agreement between the simulation results and experiment makes it a source of data to test our new *MUSCAT* procedure under the conditions of relaxed geometry.

Despite the new simulation algorithm there are some differences between the codes which are to be compared. Firstly, Fajardo *et al.* (1998 ▸) have represented the polarization state of the photon using Stokes parameters formalism with respective equations for elastic and inelastic cross sections. In *MUSCAT*, every electric vector of the photon after the scattering is determined by the appropriate geometrical transformations. The cross sections for inelastic and elastic scattering are then described by equations (10) and (12). The two methods should be equivalent because they describe the same effect but using different coordinate systems. Secondly, the approach to the scattering process is also different. Fajardo *et al.* (1998 ▸) have chosen the scattered photons directions by sampling angular probability distributions derived from the cross sections and random sampling of the scattered photon energy after that. In *MUSCAT*, the scattered photon direction is chosen by random sampling. The energy of the scattered photon is then derived using equation (8) and electron momentum sampled with the probability proportional to the given Compton profile.

Because the photon detection method has not been described in Fajardo’s article (detector size or detection angle range) we decided to use a 2 cm-diameter detector without collimation for our corresponding *MUSCAT* simulations. Also in the case of the first simulation (presented in Fig. 1 ▸) we use a free atom theoretical Compton profile of Al (Biggs *et al.*, 1975 ▸), while for the rest of the simulations, compared with experimental data, theoretical FLAPW (full-potential linearized-augmented plane-wave) Compton profiles are used.

The first simulations have been performed for comparison with Fajardo *et al.*’s results shown in Fig. 1 in their article (Fajardo *et al.*, 1998 ▸). It is a multiple scattering simulation for 60 keV linearly polarized X-rays scattered at 90° in the polarization plane (Stokes parameter *P*_1_ = −1) on a 3 mm-thick and 20 mm-diameter Al sample (incidence angle 45°).

**Figure 4:**
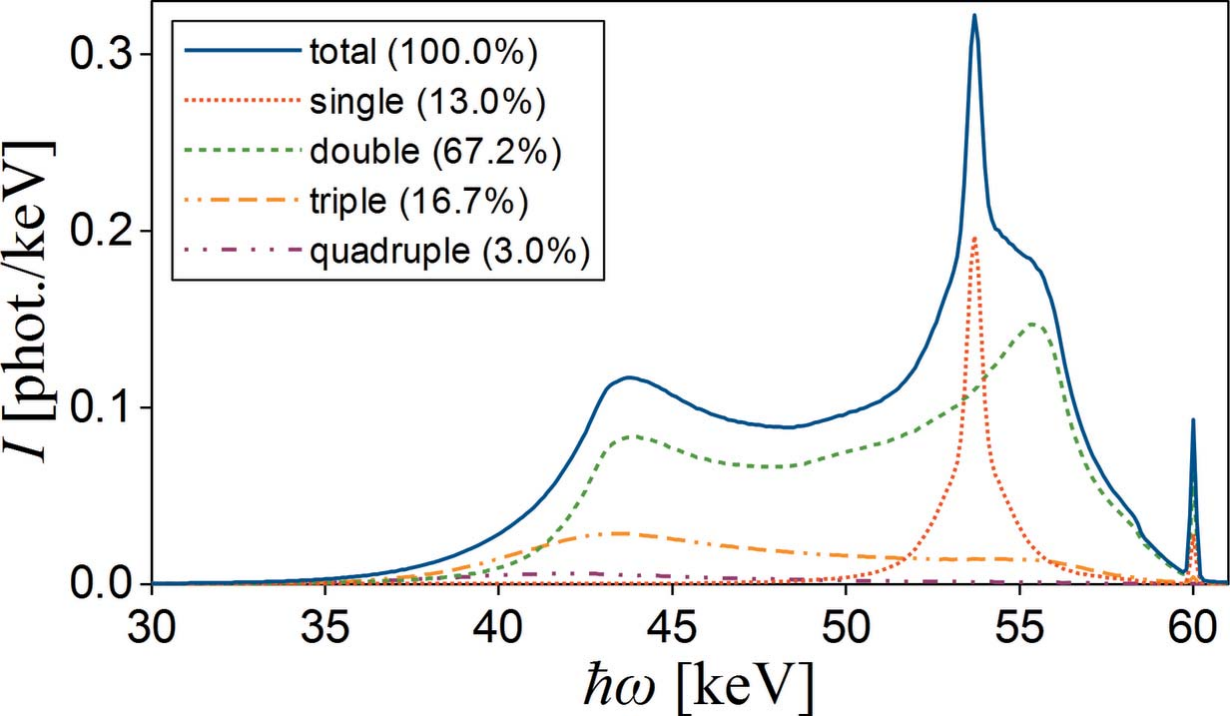
Multiple scattering simulations by *MUSCAT* for totally polarized 60 keV X-rays scattered by a 3 mm-thick Al sample at 90°. The spectrum components corresponding to different scattering levels are shown by different line colours shown in the legend together with relative intensities.

In the case of ‘perfect’ experimental conditions (no divergency of the incident beam, 100% polarization, thin sample and point detector) the single scattering intensity should disappear. Fajardo *et al.* used the real experimental parameters for this simulation, but not all of them are given directly in the description. In our simulation we used the given parameters (totally polarized beam, 90° scattering angle, sample thickness 3 mm, sample diameter 20 mm). We also assumed that the detector of diameter 2 cm is placed 1 m from the sample and the point radiation source is a distance of 50 m from the sample and is collimated to a size of 0.5 mm × 0.5 mm. The free atom Compton profile was used as an input. In this particular case, the single scattering intensity is not vanishing, it is significantly reduced, but the dominant multiple scattering can be easily observed. Simulation results by *MUSCAT* are presented in Fig. 4 ▸. The relative intensities of partial spectra are: 13% for single, 67.2% for double, 16.7% for triple and 3% for quadruple scattering, while the corresponding values obtained from Fajardo *et al.*’s simulations are: 13.1%, 66.4%, 17.2% and 3.2%. The total spectrum shape and edge around 58.5 keV resulting from the binding effect of Al *K*-shell electrons are also reproduced well.

**Figure 5:**
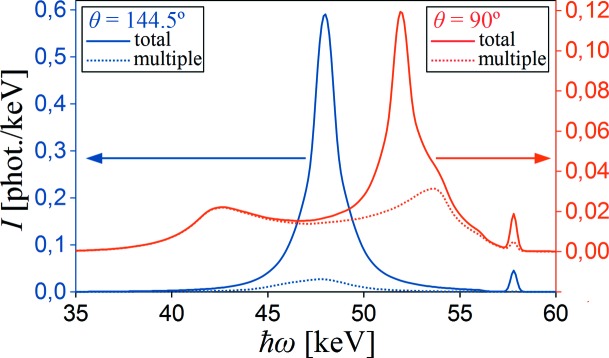
Simulated (by *MUSCAT*) spectra of 57.8 keV polarized X-rays (*P*_1_ = −0.989) scattered by a 1.5 mm-thick Al sample and registered by two detectors (resolution 0.375 keV) at different scattering angles. Total spectra intensities are shown by solid lines, multiple (up to quadruple) scattering contributions are plotted by dotted lines.

The first experimental test of the simulation procedure presented by Fajardo *et al.* (1998 ▸) is a comparison of the spectra measured at different scattering angles: 90° and 144.5°. Corresponding *MUSCAT* simulations for the same experimental parameters are shown in Fig. 5 ▸. The multiple scattering contribution to the total spectra intensity is 63% for 90° geometry and 10% for 144.5° geometry (11% in Fajardo’s simulations). The shape of spectra simulated by *MUSCAT* qualitatively agrees with those presented by the ESRF group.

**Figure 6:**
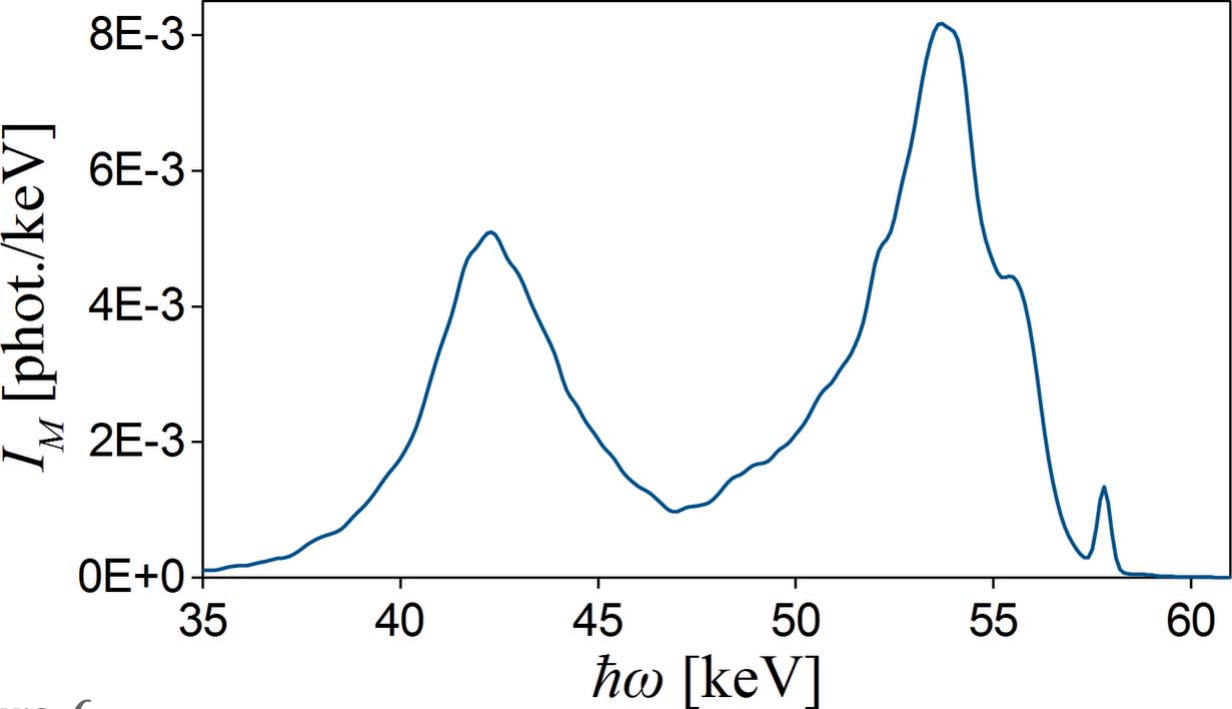
Difference between two spectra simulated by *MUSCAT* for a 57.8 keV polarized beam (*P*_1_ = −0.989), registered for 90° scattering angle by a detector of resolution 0.375 keV, for samples of the same thickness (0.5 mm) and different diameters (5 and 19 mm).

The second test is the observation of a difference between two spectra measured at 90° with a beam size of 0.5 mm × 0.5 mm, for samples with the same thickness (0.5 mm) but different diameters (5 and 19 mm), where the single scattering contribution cancels and only the difference between two multiple scattering spectra can be observed (Fig. 6 ▸). The difference spectrum shape agrees with the corresponding simulations and experimental data presented by Fajardo *et al.* (1998 ▸) (Fig. 5 ▸).

The third experimental test is also an observation of a difference in spectra measured at high scattering angle (146.5°) on samples with the same diameter but different thickness (3 and 0.5 mm).

**Figure 7:**
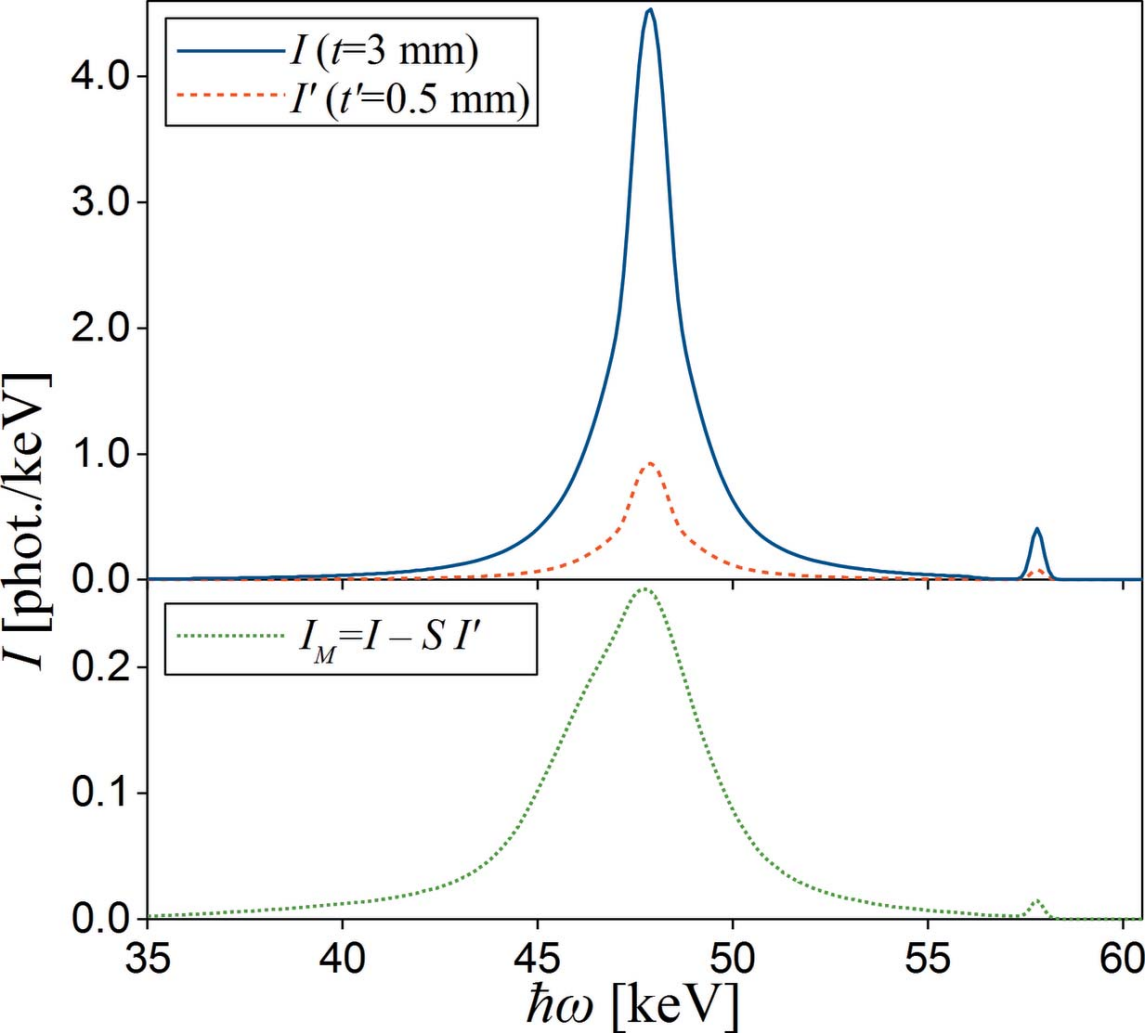
*MUSCAT* simulation results for scattering of polarized X-rays (*P*_1_ = −0.975) scattered at an angle of 146.5° on Al samples of 19 mm diameter and different thickness (0.5 and 3 mm). The upper panel shows the real simulated spectra. The lower panel shows the difference between multiple scattering components, calculated after the scaling of the spectrum simulated for the 0.5 mm sample (detailed description in the text).

In order to observe only the difference *I*_M_(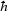
ω) between multiple scattering signals, single scattering intensities must cancel. In the case of experiments where the X-ray incidence and emission angles are fixed (like in relaxed geometry), the single scattering intensity is proportional to the effective thickness of the sample. Therefore, by multiplying the thin (0.5 mm) sample spectra *I*′(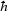
ω) by the scaling function *S*(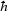
ω), the spectra can be normalized such that the single scattering intensity is the same as that of the thick (3 mm) sample spectra *I*(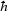
ω). Here, the *S*(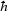
ω) function is calculated analytically as the ratio *t*_eff_/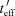, where *t*_eff_ and 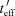 are the effective thicknesses of the thick and thin samples, respectively, and they can be calculated using equation (13) of Fajardo *et al.* (1998 ▸). The final difference between the multiple scattering signals is:
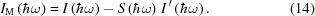
Both spectra simulated by *MUSCAT* (without scaling) and their difference (after 0.5 mm spectrum scaling) are shown in Fig. 7 ▸. The *MUSCAT* results show a good agreement with data presented by Fajardo *et al.* (1998 ▸) (Fig. 6 ▸).

## 5. Restricted geometry test   

**Figure 8:**
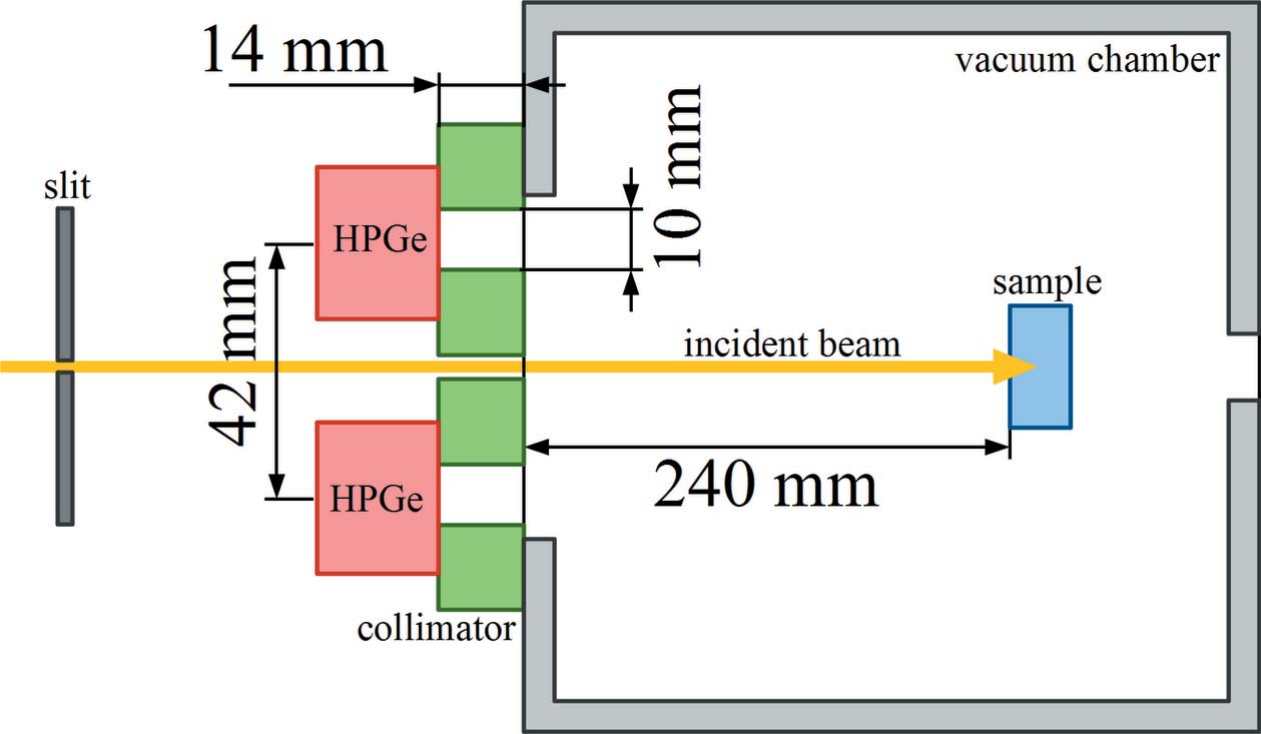
Sketch of the experimental setup cross section. Incident beam (yellow arrow) hits the sample (blue) placed in the vacuum chamber (grey). Scattered photons are registered by the HPGe detectors (red) placed after the collimation plate (green).

An experimental test of *MUSCAT* results for the restricted geometry has been performed based on the idea presented by Fajardo *et al.* (1998 ▸), with the use of samples with different thicknesses at a high scattering angle. In our experiment performed at the BL08W beamline in SPring-8 we use a 100% elliptically polarized 182.6 keV synchrotron X-ray beam size of about 0.3 mm × 0.3 mm. The corresponding polarization Stokes parameters are: *P*_1_ = −0.84, *P*_2_ = 0, *P*_3_ = 0.55. Scattered X-rays were recorded by ten HPGe detectors (resolution 0.6 keV) equally spaced around the incident beam on a circle of diameter 42 mm. The samples were placed in a vacuum chamber at a distance of 254 mm from the detector’s plane. There was a 14 mm-thick collimator and a 10 mm-diameter collimator placed in front of each detector (plate with ten holes). The scattering angle was about 175°. A sketch of the experimental setup is shown in Fig. 8 ▸. Two Al and two Cu samples with face dimensions of 20 mm × 20 mm and thicknesses of 10 mm and 1 mm were used to record four spectra of scattered photons. Background spectra were measured without a sample and subtracted from experimental data. Measured spectra were also corrected for detector efficiency.

Corresponding MSC simulations for presented geometry and all samples have been performed using *MUSCAT* up to the sixth scattering level for 10^8^ incident photons. Simulated spectra were convoluted with a Gaussian of FWHM = 0.6 keV to mimic the detector resolution. The partial spectra contributions to the total simulated intensity are given in Table 1 ▸.

**Figure 9:**
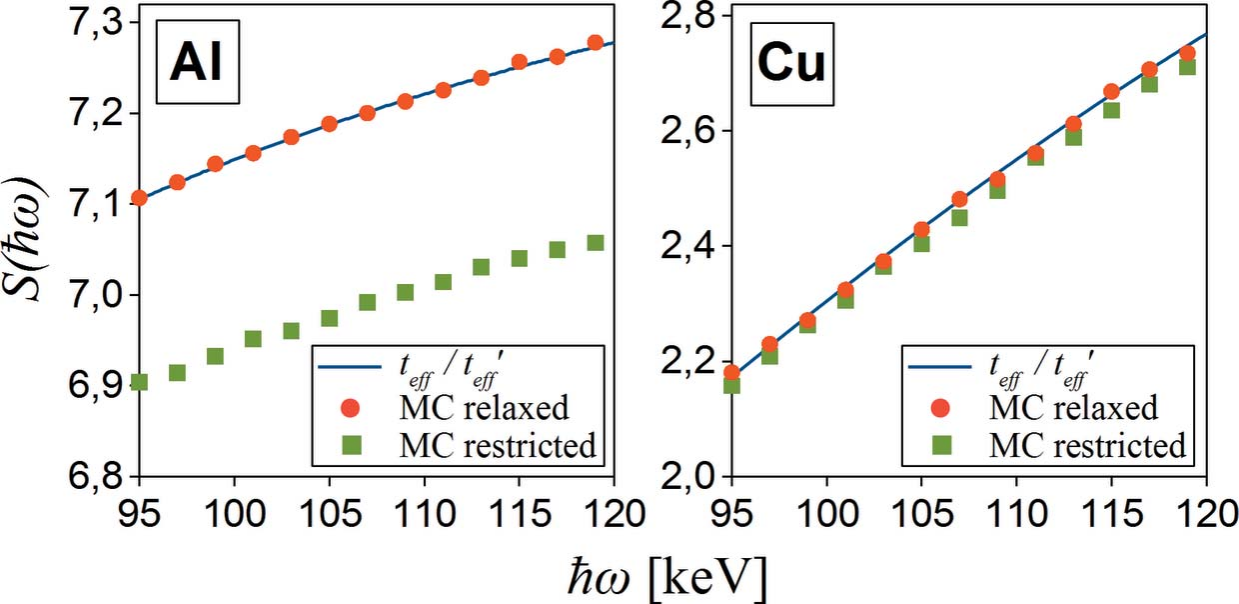
Intensity scaling functions *S*(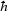
ω) for Al and Cu samples of thickness 10 mm and 1 mm calculated as the effective thickness ratio *t*_eff_/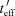 (blue solid line) and simulated by the Monte Carlo (MC) method in relaxed geometry approximation (red circles) and for real restricted geometry (green squares). The selected energy scale corresponds to the spectral range of a Compton peak in the presented experiment.

The analytically calculated scaling function *S*(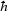
ω), like in Fajardo *et al.* (1998 ▸), can be used only for a relaxed geometry, where the X-ray incidence and emission angles are fixed. In our restricted geometry experiment (Fig. 8 ▸), however, the scattering angle has some width mainly due to the small distance between the sample and detectors. Therefore, instead of analytical calculations we employ the polynomial fit to the Monte Carlo simulated values of *S*_MC_(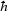
ω) in the considered energy range. Scaling functions for both Al and Cu samples (Fig. 9 ▸) were simulated for real restricted geometry (as shown in Fig. 8 ▸) and for relaxed geometry approximation (by moving the detector 100 m away from the sample).

Fig. 9 ▸ shows that the experimental geometry has a non-negligible influence on the spectrum intensity ratio. The average relative difference between the scaling function values (simulated by the Monte Carlo method) for different geometries (relaxed and restricted) is around 3% for Al and 1% for Cu within the presented energy range. The influence of the experimental geometry seems to be clear in the Al case, but in the Cu case is much smaller. This is due to the absorption difference. Photoelectric absorption in the case of Cu is still quite low at 182.6 keV energy, but the scaling function *S*(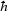
ω) is very sensitive even for the smallest absorption changes.

**Figure 10:**
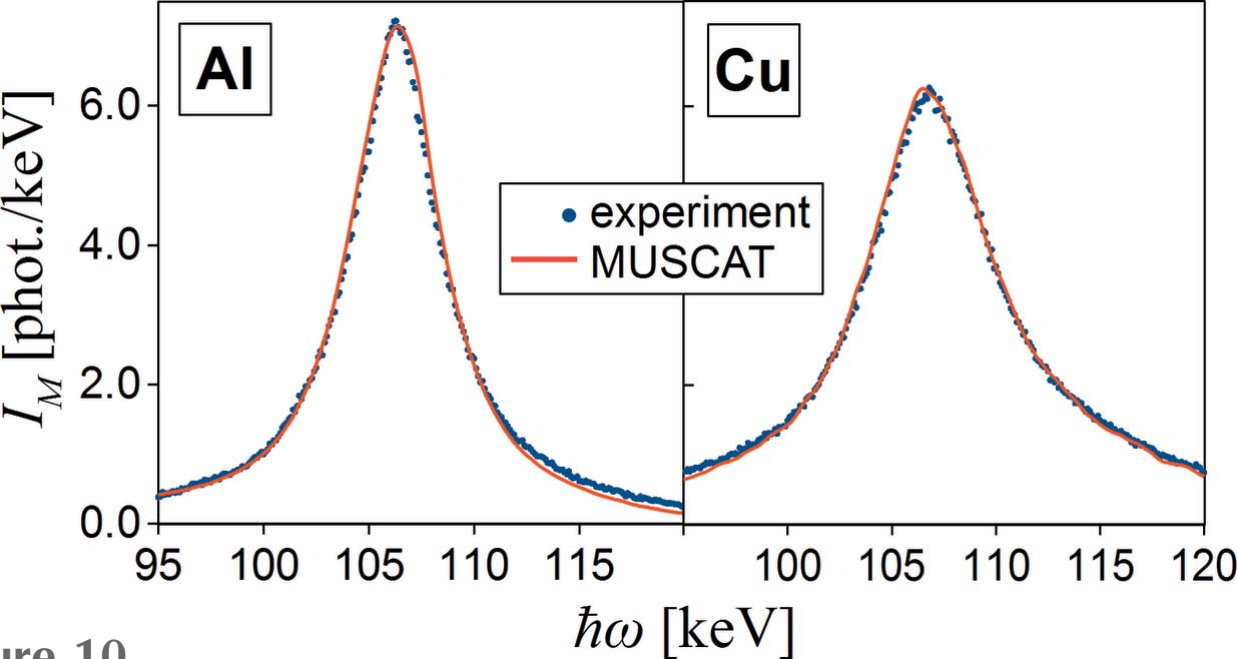
Difference between multiple scattering parts of the Compton spectra measured on thick (10 mm) and thin (1 mm) samples of Al and Cu. Experimental data are shown by points while the solid line represents the corresponding simulation results using *MUSCAT*.

In order to observe the difference in multiple scattering contributions *I*_M_(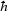
ω), measured on 10 mm- and 1 mm-thick samples [*I*(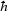
ω) and 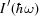, respectively] of Al and Cu, spectra for thin samples have been scaled using *S*_MC_(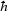
ω) as a second-degree polynomial fit to the simulated Monte Carlo data for the real restricted geometry as shown in Fig. 9 ▸:
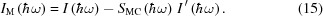
Experimental data and corresponding simulation results by *MUSCAT* are shown in Fig. 10 ▸. There is a good agreement between the simulated and experimental difference spectra although some asymmetry is observed especially in the case of Al. The most likely cause of this is the experimental background influence that cannot be measured precisely and is not completely cancelled in the difference spectrum. A small shift in the simulated and experimental peak positions may be due to the inaccuracy of the scattering angle determination in the real experimental setup.

## 6. Summary and conclusions   

The final goal of our study is to develop an efficient Monte Carlo code for high-energy photon transport in layered structure samples and build it into the procedure for multiple scattering simulations for Compton experiments with highly restricted geometry. Algorithm efficiency is crucial here because of the need to calculate photon propagation through multiple layers (say about 100). In this paper we present the first stage of our work: development of an efficient algorithm for multiple-scattered photon transport in a single material. This algorithm is built into the *MUSCAT* program which can simulate a wide range of experimental geometries that are typically used in Compton experiments, including highly restricted ones.

The idea of multiple scattering simulations by the Monte Carlo method in Compton spectroscopy is not new, but it has been sufficiently developed and experimentally verified only in 1998 by the group from ESRF (Fajardo *et al.*, 1998 ▸). Details of the algorithm are not described herein, but we found some significant differences compared with the *MUSCAT* program.

In order to check the *MUSCAT* program under the restricted geometry, we have performed a new experiment with the use of elliptically polarized high-energy (182.6 keV) synchrotron radiation. In the case of a high scattering angle (175°), polarization of the beam has no noticeable effect on registered spectra, but it has been shown that some geometric restrictions due to the short distance between the sample and detector are significant. The intensity ratio of single-scattered photons spectra for thin (1 mm) and thick (10 mm) samples of Al and Cu represented by the scaling function *S*_MC_(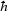
ω) differ from analytically calculated values in relaxed geometry approximation by about 3% for Al and 1% for Cu. In the case of stronger restrictions, this effect will be much more significant. The *MUSCAT* simulation results proved to be in good agreement with the experimental data for both materials.

It has been shown that the new program *MUSCAT* for high-energy photon transport in matter and multiple scattering simulations gives results which are consistent with various experimental data and another independently developed program. Therefore, the *MUSCAT* code can be used for further research and implementation of photon transport through multilayered structure samples.

In its current shape the *MUSCAT* program is available for use by other researchers after contact with the corresponding author (Marek Brancewicz) at brancew@spring8.or.jp. Since the full manual has not been prepared yet, the author will provide all necessary support to run the simulation procedure properly.

## Figures and Tables

**Table 1:** Partial spectra intensities (in % of total intensity) for the experiment simulated by *MUSCAT*. All spectra intensities have been calculated in the energy range 94.2–118.9 keV, which is equivalent to a Compton profile momentum range from −10 to 10 a.u

	Partial spectra intensity (%)					
Sample (thickness)	1st	2nd	3rd	4th	5th	6th
Al (10 mm)	73.89	22.90	2.93	0.26	0.02	0.00
Al (1 mm)	92.95	6.84	0.21	0.01	0.00	0.00
Cu (10 mm)	62.41	27.65	7.75	1.78	0.34	0.06
Cu (1 mm)	83.85	14.60	1.43	0.12	0.01	0.00
